# Feature selection and classification of urinary mRNA microarray data by iterative random forest to diagnose renal fibrosis: a two-stage study

**DOI:** 10.1038/srep39832

**Published:** 2017-01-03

**Authors:** Le-Ting Zhou, Yu-Han Cao, Lin-Li Lv, Kun-Ling Ma, Ping-Sheng Chen, Hai-Feng Ni, Xiang-Dong Lei, Bi-Cheng Liu

**Affiliations:** 1 Institute of Nephrology, Zhong Da Hospital, Southeast University School of Medicine, Nanjing, Jiangsu, China; 2 CT Bioscience CO. LTD, Changzhou, Jiangsu, China

## Abstract

Renal fibrosis is a common pathological pathway of progressive chronic kidney disease (CKD). However, kidney function parameters are suboptimal for detecting early fibrosis, and therefore, novel biomarkers are urgently needed. We designed a 2-stage study and constructed a targeted microarray to detect urinary mRNAs of CKD patients with renal biopsy and healthy participants. We analysed the microarray data by an iterative random forest method to select candidate biomarkers and produce a more accurate classifier of renal fibrosis. Seventy-six and 49 participants were enrolled into stage I and stage II studies, respectively. By the iterative random forest method, we identified a four-mRNA signature in urinary sediment, including TGFβ1, MMP9, TIMP2, and vimentin, as important features of tubulointerstitial fibrosis (TIF). All four mRNAs significantly correlated with TIF scores and discriminated TIF with high sensitivity, which was further validated in the stage-II study. The combined classifiers showed excellent sensitivity and outperformed serum creatinine and estimated glomerular filtration rate measurements in diagnosing TIF. Another four mRNAs significantly correlated with glomerulosclerosis. These findings showed that urinary mRNAs can serve as sensitive biomarkers of renal fibrosis, and the random forest classifier containing urinary mRNAs showed favourable performance in diagnosing early renal fibrosis.

Chronic kidney disease (CKD) is a worldwide public health problem, affecting 12% of all adults in the United States and 10.8% in China^1^,^2^. Renal fibrosis is a common pathological pathway of progressive CKD, which is characterized as a relentless deposition of extracellular matrix (ECM) with concomitant loss of the parenchyma.

Renal biopsy is the gold standard for measuring fibrosis. Tubulointerstitial fibrosis (TIF) and glomerular sclerosis (GS) quantification are considered the best available pathological markers of chronic kidney injury^3^. Unfortunately, such invasive examination may cause bleeding and other complications, which impede its repeated application. In clinical practice, kidney function estimations such as serum creatinine (SCr) and SCr-based estimated glomerular-filtration rate (eGFR) measurements are most widely used to evaluate renal fibrosis. However, SCr usually changes little at the onset of fibrosis^4^. Although demonstrated to provide a good estimation of kidney function, eGFR calculated by the Cockcroft–Gault or MDRD (Modification of Diet in Renal Disease) formula is less accurate in early kidney disease and underestimates the GFR of healthy individuals^5^. Thus, it is imperative to develop validated biomarkers to monitor early fibrosis with minimal damage.

Recently, analysing the mRNAs of urinary sediment has raised great interest as one feasible strategy. Urinary mRNAs have been reported to increase sharply at an early stage in a rat model of human diphtheria toxin receptor progression^6^. Vimentin, NKCC2, E-cadherin, and 18S rRNA mRNA in urinary sediment correlated with the severity of renal fibrosis in human kidney allografts^7^. We demonstrated that urinary podocalyxin, CD2-AP, α-actin4, and podocin mRNAs correlated with SCr in patients with diabetic nephropathy (DN)^8^. Recently, microarrays have been used as a high-throughput screening platform to discover potential mRNA biomarkers. Using targeted microarrays, our previous study showed that urinary vimentin mRNA was significantly upregulated in moderate-to-severe fibrosis^9^. However, whether urinary mRNAs can efficiently identify patients with renal fibrosis in the context of CKD has not been investigated yet.

Another problem is that datasets generated by microarrays are often noisy, multicollinear, and high dimensional, which make it difficult to process. Machine learning is a subfield of computer science that evolved from artificial intelligence. Machine learning can be used to process much more complex data than traditional statistical methods and make predictions with higher accuracy^10^. Random forest (RF) methods, constructed from decision tree predictors, represent one of the most prevalent supervised machine learning methods, which was first introduced by Breiman in 2001^11^. RF methods return measures of variable importance and have superior performance with respect to the problems that microarray data bring, making it well suited for microarray analysis^12^. An empirical study by Archer *et al*. showed that RF is a robust method for making an accurate classifier and evaluating the discriminative ability of individual predictors in classification problems^13^.

Patients who undergo renal biopsy are usually at the early stages of CKD, which is the ideal target population. The primary objective of this two-stage study was to test and validate the hypothesis that mRNAs from urinary sediment could provide useful information of early renal fibrosis. To our knowledge, this is the first machine-learning analysis of the diagnostic performance of urinary mRNAs in renal fibrosis. By iterative random forest analysis of a targeted microarray, we aimed to discover a panel of mRNAs and develop a more powerful classifier for improved diagnosis of renal fibrosis.

## Results

### Characteristics of the study population

Sixty-two biopsy-proven CKD patients and 14 healthy participants were included in the stage-I study. The primary diseases of the enrolled patients included IgA nephropathy (IgAN, n = 39), membranous nephropathy (MN, n = 10), minimal change disease (MCD, n = 5), non-IgA mesangioproliferative glomerulopathy (non-IgA MsPGN, n = 1), and focal segmental glomerulosclerosis (FSGS, n = 7).

Forty-one biopsy-proven CKD patients and 8 healthy participants, who were screened according to the same criteria used for stage I, were enrolled in the validation set (Table 1). Primary glomerulonephritis was still the leading cause of CKD, and 16 IgAN, 7 non-IgA MsPGN, 3 MCD, 3 MN, 1 FSGS, 1 IgM, and 1 C1q nephropathy cases were found. Other causes of CKD included lupus nephritis (n = 3), DN (n = 2), Alport’s syndrome (n = 2), and ANCA-associated vasculitis (n = 3). The basic clinical characteristics of all participants, with or without TIF, are shown in Table 1.

### Four mRNAs were identified as important features of TIF by RF

**Figure 1:**
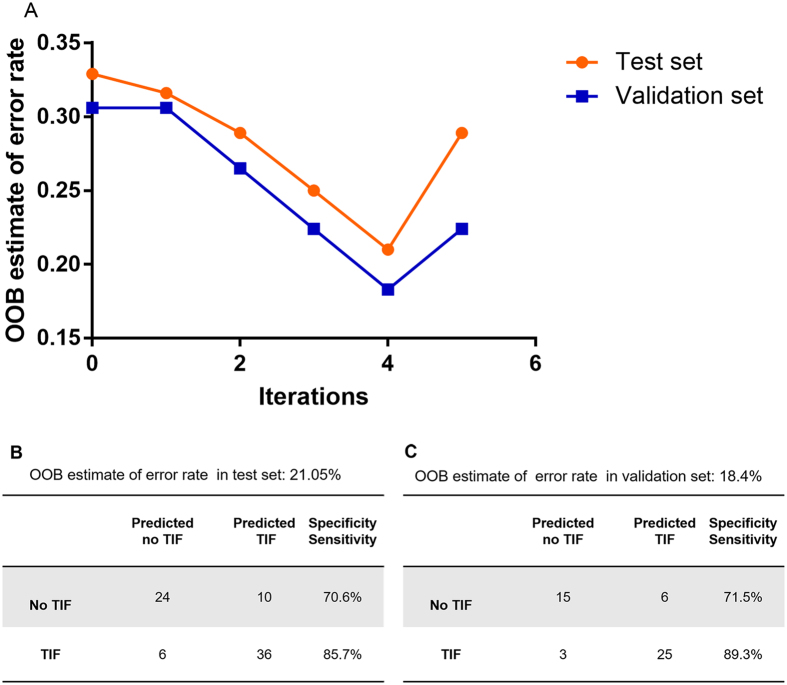
Feature selection and classification by iterative random forest. (**A**) the changes of OOB errorestimates during iterations in the test set and validation set. (**B,C**) OOB error estimates and confusion matrices for prediction of TIFby selected mRNAsin test set (**B**) and validation set (**C**).

As shown in Fig. 1, the initial out-of-bag (OOB) estimates of the error rates were 0.329 and 0.306 in the test set and validation set, respectively. After the first four iterations, the OOB error decreased as the noisy mRNAs were eliminated. The error rates of both sets rebounded at the fifth iteration; thus, the “early stop” strategy was applied and the final OOB error was 0.210 in the test set and 0.183 in the validation set. Consequently, four mRNAs including TGFβ1 (TGB1), MMP9, TIMP2, and vimentin (VIM) were identified as important features of TIF by RF (Fig. 1).

### Use of four selected mRNAs to diagnose TIF with a high sensitivity

**Figure 2:**
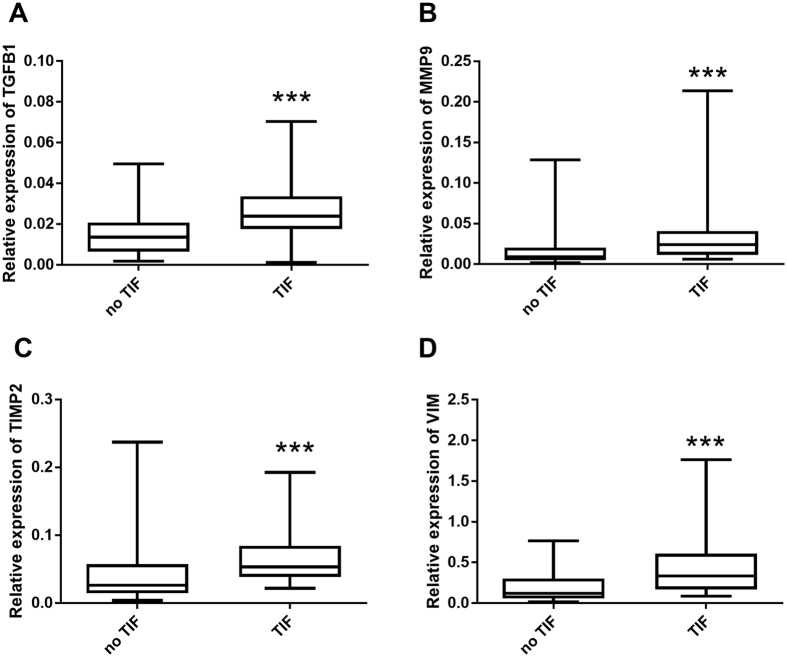
The differential expression of the selected four mRNAs between the no TIF group and TIF group by Mann–Whitney test, box plots show the lower 95% CI, 25th, median, 75th, and the upper 95% CI values of the mRNA relative expression. (**A–D**) the relative expressions of TGFβ1 mRNA (**A**: 0.016 versus 0.027, p < 0.001), MMP9 mRNA (**B**: 0.040 versus 0.021, p < 0.001), TIMP2 mRNA (**C**: 0.052 versus 0.072, p < 0.001) and vimentin mRNA (**D**: 0.20 versus 0.47, p < 0.001)were significantly higher in TIF group than no TIF group. ***p < 0.001.

**Figure 3:**
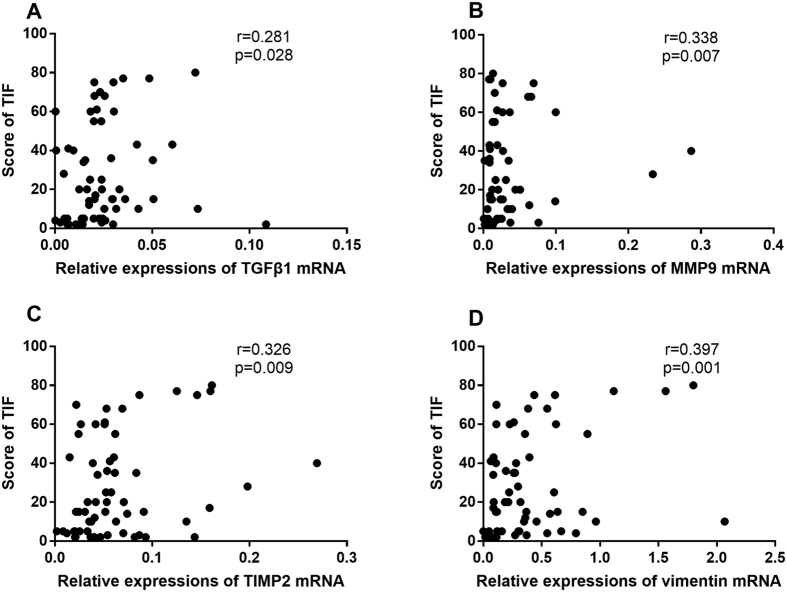
The correlation between the score of TIF and selected mRNAs by Spearman’s analysis with healthy participants excluded. (**A–D**) the score of TIF positively correlated with the relative expression of TGFβ1 (**A**), MMP9 (**B**), TIMP2 (**C**) and vimentin (**D**) mRNAs significantly.

We first examined the basic statistical associations between the selected mRNAs and TIF in the test set. The relative expression levels of the four mRNAs in the TIF group were significantly higher than those in the group without TIF (Fig. 2). In addition, the TIF score (the healthy participants were not included in this analysis) and relative expression levels of TGFβ1 (r = 0.281, p = 0.028), MMP9 (r = 0.338, p = 0.007), TIMP2 (r = 0.326, p = 0.009), and vimentin (r = 0.397, p = 0.001) were significantly correlated (Fig. 3).

**Figure 4:**
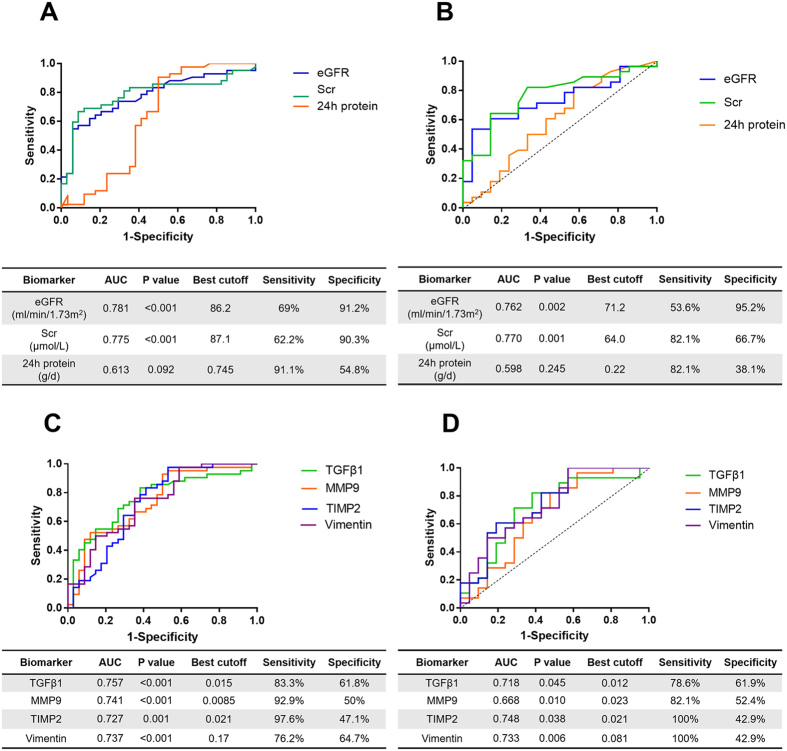
The diagnostic power of individual mRNAs and routine parameters in TIF by ROC curve analyses. (**A,B**) the performance of eGFR, SCr and 24 h protein in diagnosing TIF in test set (**A**) and validation set (**B**). (**C,D**) the performance of TGFβ1, MMP9, TIMP2 and vimentin mRNAs in diagnosing TIF in test set (**C**) and validation set (**D**).

Then, we assessed the individual diagnostic power of the four mRNA biomarkers in the test set by receiver-operating characteristic (ROC) curve analysis. As shown in Fig. 4, all biomarkers showed moderate performance in discriminating TIF, with areas under the ROC curve (AUCs) ranging from 0.727 to 0.757. The best cut-off of the four mRNAs yielded good sensitivity (0.762 to 0.976) but poor specificity (0.471 to 0.647), indicating the screening value of these biomarkers. Their performance was further validated in the stage-II study (AUCs between 0.668 and 0.748) (Fig. 4). In the test set, eGFR and SCr yielded better overall performance than did the individual mRNAs with AUCs of 0.781 (95% CI, 0.672–0.890, p < 0.001) and 0.775 (95% CI, 0.669–0.881, p < 0.001), respectively. The best cut-off values for both eGFR and SCr yielded excellent specificity (0.912 for eGFR and 0.903 for SCr), but poor sensitivity (0.690 for eGFR and 0.622 for SCr). In contrast, 24 urine proteins failed to discriminate TIF, both in the test set (AUC = 0.613, p = 0.092) and validation set (AUC = 0.598, p = 0.245).

### mRNA classifier trained by RF outperformed SCr and eGFR in diagnosing TIF

As shown in Table 2, the best cut-offs of SCr and eGFR obtained from the test set (87.1 μmol/L for SCr and 86.2 ml•min•1.73 m^2^ for eGFR) had diagnostic accuracy of 0.673 (sensitivity of 0.536 and specificity of 0.857 for SCr) and 0.694 (sensitivity of 0.607 and specificity of 0.810 for eGFR), respectively. The diagnostic accuracy of the individual mRNA ranged from 0.633 to 0.693. However, the combined mRNA classifier outperformed kidney function parameter testing in the validation set, yielding an accuracy of 0.796 (sensitivity of 0.929 and specificity of 0.619). We further tested whether the RF classifier consisted of combined mRNAs and kidney function parameters had better performance. Impressively, the accuracy was further elevated to 0.877 (sensitivity of 0.964 and specificity of 0.762) and 0.857 (sensitivity of 0.964 and specificity of 0.714) when eGFR and SCr were added, respectively.

### Urinary mRNAs correlated with glomerular sclerosis

**Figure 5:**
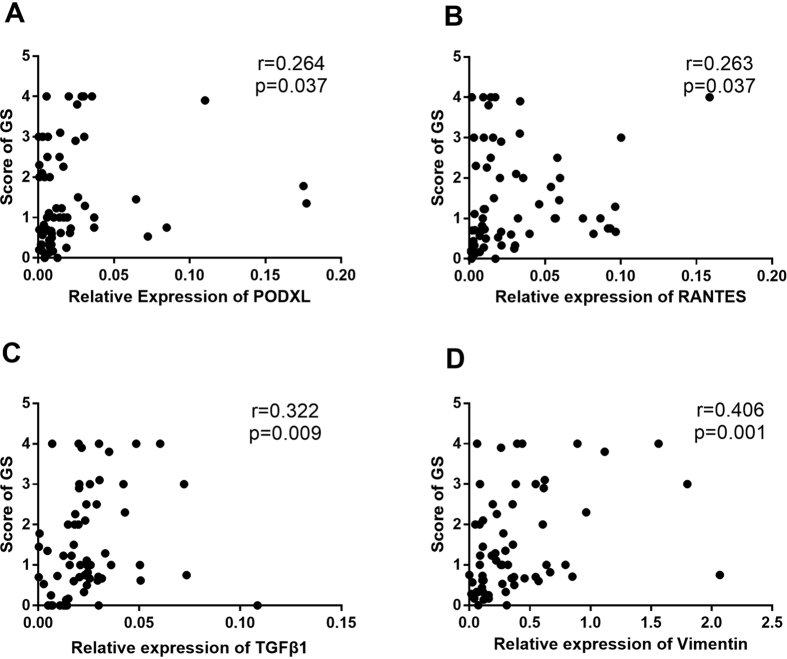
The correlations between the score of GS and selected mRNAs by Spearman’s analysis with healthy participants excluded. (**A–D**) the score of GF positively correlated with the relative expression of PODXL (**A**), RANTES (**B**), TGFβ1 (**C**) and vimentin (**D**) mRNAs significantly.

Based on the % IncMSE values of RF regression, we identified vimentin, TGFβ1, RANTES, and PODXL as the most important genes associated with GS in the test set. The GS score (the healthy participants were not included in this analysis) and the relative mRNA-expression levels of PODXL (r = 0.264, p = 0.037), RANTES (r = 0.263, p = 0.037), TGFβ1 (r = 0.322, p = 0.009), and vimentin (r = 0.406, p = 0.001) (Fig. 5) were significantly correlated.

## Discussion

A major challenge for early detection of renal fibrosis is the lack of early and noninvasive biomarkers. However, previous evidence revealed that kidney function parameters were suboptimal for detecting early fibrosis owing to the compensatory effect^14^. Moreover, these markers reveal limited information regarding the underlying molecular mechanism. Recent data have shown that measuring transcriptional differences in urine sediment from patients with CKD may provide a novel means for early and sensitive diagnosis^7^,^8^,^9^.

As microarray data is high-dimensional and reflects gene–gene interactions, univariate selection methods may generate less accurate classifiers and fail to capture these interactions^15^. In this study, we applied RF, a novel and powerful gene selection strategy, to discover renal fibrosis biomarkers.

Here, we identified a four-mRNA signature in urinary sediment including TGFβ1, MMP9, TIMP2, and vimentin, which were important features of TIF. We showed that these four mRNAs could individually serve as sensitive diagnostic biomarkers for TIF. We also found that a classifier containing all four mRNAs had favourable performance in diagnosing TIF. Impressively, the combined classifiers outperformed SCr and eGFR in the validation set, with excellent sensitivity. We also investigated correlations between urinary mRNAs and GS severity by RF regression. As a secondary result, four mRNAs showed significant correlations with the GS score. These findings extended our previous findings on the diagnostic value of urinary mRNAs in renal fibrosis.

Far from being a simple disposition of collagen, renal fibrosis is modulated by a complex signalling network^16^. The role of TGFβ1 in renal fibrosis has been well established^17^. Accumulating evidence has shown that both circulating and urinary TGFβ1 levels can serve as biomarkers for CKD^18^,^19^. Recently, TGFβ1 mRNA expression in urinary sediment was found to correlate with the degree of tubulointerstitial scarring in a small scale observational study^20^. Our study further tested the diagnostic value of urinary TGFβ1 mRNA levels on a larger sample. A vast array of additional molecules has also been demonstrated to serve modulatory roles. Metal matrix proteinases (MMPs) and tissue inhibitor of metalloprotease (TIMP) proteins are recognized as the major cellular factors mediating matrix turnover^16^. In addition to ECM proteins, growth factor receptors and cell-adhesion molecules are also MMP substrates. Friese *et al*. reported that MMP-9 was up-regulated in hypertension and hypertensive end-stage kidney disease (ESKD)^21^. Recent findings have implied that TEC, MMP-9, and TIMP-2 expression can be upregulated by TGF-β in disease models^22^,^23^. We previously reported that the expression of MMP9 mRNA was strongly correlated with kidney function parameters^9^. Here, we further showed that these two mRNAs could serve as TIF biomarkers. Vimentin, a marker for EMT (epithelial-mesenchymal transition), has been demonstrated to be overexpressed in tubular cells during renal fibrosis^24^. Lee *et al*. found that a four-gene signature of mRNAs, including vimentin, was a predictor for renal fibrosis in human kidney allografts^7^. Data from our previous study revealed that vimentin mRNA detection enables good discriminative power of moderate to severe TIF. In this study, we also found that vimentin mRNA was a sensitive diagnostic marker of TIF. Although the overall performance of every single urinary mRNA markers was not superior to kidney function parameters in this analysis, ROC curve analysis showed that these markers all had better sensitivity. In fact, kidney function parameters changed little at the onset of fibrosis^4^,^5^. So urinary mRNAs can make up for the lack of sensitivity of current parameters and serve as screening markers for renal fibrosis. Moreover, kidney function parameters are also affected by non-renal factors such as prerenal ischemia and postrenal obstruction^5^. The value of urinary mRNAs in differential diagnosis needs further investigation.

No single biomarker alone is considered to be sensitive and specific enough for fibrosis screening. One possible reason is that a common pathway may not be involved in all CKD patients^25^. A previous report by Zeisberg *et al*. showed that renal fibrosis also occurs in a TGF-β signaling-independent manner^26^. Moreover, single-molecule measurements might have larger variations, which would make the associated predictions unstable. Our results showed that the combined mRNAs outperformed single mRNAs and kidney function parameters in detecting TIF, supporting the possibility that combined biomarkers could yield more accurate classification. In particular, when combined with kidney function parameters, the mRNA classifiers trained by RF yielded excellent accuracy of classification.

The main strengths of our study include: (1) the targeted microarray was constructed for high throughput analysis of urinary mRNAs; (2) strong methodology (iterative random forest) was applied to select features and generate a classifier that had a favorable performance in diagnosing TIF. (3) a two-stage study design was adopted to further validate the performance of biomarkers in diagnosing TIF. Our study has some limitations. First, the sample size of the validation set was relatively small, and a larger study is needed to further confirm our conclusions. Second, the constituents of urinary sediment in enrolled CKD patients may also interfere with the diagnostic performance of mRNAs. Urinary cell-specific mRNAs are expected to better reflect kidney injury. Third, to test the screening value of urinary mRNA biomarkers in renal fibrosis, we also included several healthy participants who did not undergo renal fibrosis and classified them into the no-fibrosis group.

In summary, we showed that among the target genes examined, four fibrosis- associated mRNAs in urinary sediments can serve as sensitive predictors of TIF. Combined classifier showed excellent sensitivity and better overall diagnostic performance than eGFR and SCr. Large-scale studies are needed to confirm the utility of urinary mRNA biomarkers for diagnosing renal fibrosis and predicting CKD progression.

## Methods

### Trial design

This two-stage study was approved by the Ethical Committee of Zhong Da Hospital (Southeast University). All participants provided written, informed consent. All methods were performed in accordance with the relevant guidelines and regulations. The stage-I study (test set) was conducted from September 2012 to September 2014, and the stage-II study (validation set) was conducted from October 2014 to February 2015. Patients with biopsy-proven CKD were included in this study, and their clinical data were collected. The eGFR was calculated according to modified MDRD equations. The exclusion criteria were as follows: a) patients younger than 18 years old; b) patients with acute kidney injury, chronic liver disease, cardiovascular disorders, urinary tract infections, or cancer; and c) administration of immunosuppressive medications.

A group of age- and gender-matched healthy volunteers from the Zhong Da Hospital Health Care Center was also included in this study, all of whom met the following criteria: (1) no record of abnormal renal function (eGFR < 90 mL•min•1.73 m^2^); (2) normal routine urinalysis, ACR (albumin-creatinine ratio), and 24-h urinary protein test results; (3) no record of hypertension, diabetes, hyperlipidaemia, or hyperuricaemia; (4) no family history of kidney diseases.

### Construction of the urinary mRNA microarray

We designed an mRNA microarray containing 61 genes participating in the well-accepted molecular mechanism of renal fibrosis. PRIMER 5 software was used to design the qPCR primer sets. Six reference genes (GAPDH, B2M, OAZ1, RPL27, HRPT1, and ACTB) were included in the microarray to normalize the transcription levels. Furthermore, we also included a genomic DNA control (GDC) to control for DNA contamination. The list of included genes is shown in Table 3. The primer sequences of all included genes are shown in the supplemental material.

### Urine samples and mRNA measurements

First morning urine samples obtained at the time of kidney biopsy were centrifuged at 3,000 × *g* for 20 min at 4 °C within 2 h after sample collection. Then, the obtained urinary sediments were resuspended in 1.5 ml DEPC-treated PBS and centrifuged at 12,000 × *g* for 5 min at 4 °C. One millilitre of RNAiso Plus (Takara, Life Technologies) was added to preserve total RNA, and the samples were stored at −80 °C until use. Total RNA was extracted according to the manufacturer’s protocol (Ambion, Life Technologies). We measured the RNA concentrations using a NanoDrop 2000 (Thermo) based on the relative absorbance ratio at 260/280, and the RNA purity was also assessed by agarose gel electrophoresis. The qualified RNA samples were stored at −80 °C until use.

We used the kit from Invitrogen to reverse transcribe the total RNA (Invitrogen, Life Technologies), which was stored at −20 °C until use. mRNA-expression levels were quantified by real-time quantitative polymerase-chain-reaction (qPCR) assays in an ABI PRISM7700 system (Applied Biosystems). The thermocycling conditions were set as follows: 95 °C for 10 min, followed by 40 cycles of 15 s at 95 °C and 60 °C for 1 min. Dissociation curves and melting temperatures were recorded and relative mRNA expression levels were calculated by the ΔΔCt method^27^.

### Evaluation of renal fibrosis

Periodic acid Schiff (PAS) and Masson trichrome staining were used to determine the severity of GS and TIF, respectively. Two experienced pathologists (P.S. Chen and H.F. Ni) were blinded to the microarray data and scored the severity of renal fibrosis. For GS, a semiquantitative scoring system was used, as described previously by Raji *et al*.^28^. In specimens containing at least 20 glomeruli, each glomerulus was graded from 0 to 4 according to the percentage of fibrotic area (0 for 0%; 1 for 1–25% affected glomerular area; 2 for 26–50% affected glomerular area; 3 for 51–75% affected glomerular area; 4 for 76–100% affected glomerular area). The final score was a weighted average of all grades obtained. The percentage of fibrotic area in tubulointerstitium was recorded as the TIF score, and its grade was based on the following well-accepted rules: grade 0, no more than 5% fibrotic area; 1, 6–25% fibrotic area; 2, 26–50% fibrotic area; and 3, >50% fibrotic area. A grade of 0 was considered to reflect the absence of TIF. Participants showing grades of 1–3 were combined into the TIF group. All healthy participants were classified in the no-TIF group.

### Statistical analysis

Our analyses of the microarray database involved the following steps: (1) select important TIF features by iterative RF; (2) assess the individual diagnostic power of selected mRNAs for TIF, as well as their correlation with scores of TIF, in the test set; (3) validate the individual diagnostic power of selected mRNAs and routine parameters for TIF in the validation set; (4) assess the diagnostic power of the classifiers trained by RF for TIF and compare them with those of eGFR and SCr in the validation set; (5) select important features of GS by iterative RF regression and assess their correlation with GS scores.

### Iterative RF method

In the test set, iterative RF was performed to classify cases with and without TIF, using the randomForest package (from A. Liaw and M. Wiener) in R software (version i386 3.2.4)^29^. After each iteration, mRNAs showing the smallest mean decrease in accuracy were discarded, and a new RF with a lower OOB error rate was constructed. Data from a previous study have shown that the OOB estimate is as accurate as using a test set of the same sample size as the training set^11^. Although RF was more resistant to over-fitting than a support vector machine or artificial neural network, the validation set was involved in the same process simultaneously and an “early-stop” strategy was applied to prevent “over-fitting”^12^. The final set of mRNAs with smallest estimated OOB was identified as important features of TIF. For regression, the % IncMSE was used as a parameter for determining the importance of each mRNA, and the same iterative process was undertaken.

### Other statistical methods

SPSS 18.0 software was used for all additional statistical analyses. The Kolmogorov–Smirnov test was used to determine the normality of the data. Numeric results subjected to normal distribution were presented as the mean ± SD. Non-normal numeric results were presented as quartiles. Student’s t-test was applied to compare the means of normal data. Otherwise, the Mann–Whitney test was applied. The correlation between gene-expression levels and pathological parameters was analysed by Spearman’s rank-order test. The diagnostic performance of single biomarker was evaluated by generating ROC curves. The AUC was used to assess the overall discriminative power. An AUC of 0.6–0.7 was considered as poor, 0.7–0.8 was moderate, 0.8–0.9 was good, and >0.9 was excellent. The best cut-offs were determined by selecting the data points that maximized the sum of specificity and sensitivity on the ROC curve. Two-tailed P values of <0.05 were considered statistically significant.

## Additional Information

**How to cite this article**: Zhou, L.-T. *et al*. Feature selection and classification of urinary mRNA microarray data by iterative random forest to diagnose renal fibrosis: a two-stage study. *Sci. Rep.* **7**, 39832; doi: 10.1038/srep39832 (2017).

**Publisher's note:** Springer Nature remains neutral with regard to jurisdictional claims in published maps and institutional affiliations.

## Supplementary Material

Supplementary Information

## Figures and Tables

**Table 1:** Basic characteristics of the participants with and without TIF in stage-I and stage-II studies.

	Test Set				Validation Set			
	Total(n = 76)	TIF(n = 42)	no TIF(n = 34)	P value	Total(n = 49)	TIF(n = 28)	no TIF(n = 21)	P value
Age	41.4 ± 14.4	39.1 ± 11.9	43.4 ± 16.0	0.198	39.7 ± 13.8	40.1 ± 14.1	39.1 ± 13.6	0.811
Gender (male/female)	40/36	24/18	16/18	0.381	27/22	42720	42684	0.74
SCr (μmol/L)	78.3 [61.0–107.9]	100.1 [68.8–131.0]	63.3 [57.0–80.0]	<0.001*	68 [59–116]	96.5 [65.0–151.0]	60 [54.5–70.0]	0.001*
eGFR (ml/min/1.73 m^2^)	88.6 ± 33.7	74.6 ± 34.8	105.9 ± 22.9	<0.001*	93.5 ± 41.0	79.9 ± 43.2	111.7 ± 30.1	0.006*
24 h urinary protein (g/d)	2.17 [0.59–4.44]	2.48 [1.26–3.77]	1.21 [0.10–4.72]	0.092	1.2 [0.08–3.09]	1.61 [0.44–3.38]	0.97 [0.03–2.85]	0.245
SBP (mmHg)	132.1 ± 14.6	136.1 ± 16.1	127.2 ± 10.7	0.051	132.3 ± 15.3	133.8 ± 17.5	130.2 ± 12.1	0.43
DBP (mmHg)	81.4 ± 8.7	82.9 ± 9.8	79.5 ± 6.6	0.177	80.5 ± 8.5	81.9 ± 9.2	78.5 ± 7.1	0.171

Values for continuous variables are given as mean ± SD or median [25th–75th percentile].

**Table 2:** The diagnostic accuracy, sensitivity and specificity of individual and combined biomarkers in the validation set.

Classifer	Accuracy	Sensitivity	Specificity
4 mRNAs	0.796	0.929	0.619
4 mRNAs + eGFR	0.877	0.964	0.762
4 mRNAs + SCr	0.857	0.964	0.714
eGFR	0.694	0.607	0.810
Scr	0.673	0.536	0.857
TGFβ1	0.693	0.714	0.666
TIMP2	0.673	1.0	0.238
MMP9	0.678	0.964	0.296
Vimentin	0.633	0.750	0.476

**Table 3:** A display of 61 genes in the targeted microarray.

1	2	3	4	5	6	7	8	9	10	11	12	13	14	15	16
ACE	AGT	BMP7	C1GALT1	C1GALT1C1	CASP3	CCL2	CCL4	CCL5	CDH1	COL3A1	COL4A3	CTGF	EGF	FABP1	FN1
HAVCR1	HGF	ICAM1	IGF1	IL10	IL18	IL1B	IL6	IL8	ILK	LCN2	MMP2	MMP7	MMP9	NFKB2	NLRP3
NPHS1	NPHS2	PDGFA	PDGFB	PLAUR	PODXL	REN	S100A4	SERPINE1	SMAD2	SMAD3	SMAD4	SMAD7	SNAI1	SNAI2	ST6GALNAC2
SYNPO	TF	TFRC	TGFB1	TGFB2	TIMP1	TIMP2	TNF	TNFSF13	TP53	TWIST1	VEGFA	VIM			
