# Supplementary material for: Feature selection and classification of urinary mRNA microarray data by iterative random forest to diagnose renal fibrosis: a two-stage study
Source: Sci Rep. 2017 Jan 3;7:39832. doi: 10.1038/srep39832 (PMC5206620; doi:10.1038/srep39832)
Supplement: Supplementary Information [file srep39832-s1.doc]

**Feature selection and classification of** **urinary mRNA microarray data by** **iterative random forest to diagnose renal fibrosis: a two-stage study**

**Le-Ting Zhou****^1^**, **Yu-Han** **Cao^1^**, **Lin-Li Lv^1^**, **Kun-Ling Ma^1^**, **Ping-Sheng Chen^1^**, **Hai-Feng****Ni^1^, Xiang-Dong Lei^2^ & Bi-Cheng** **Liu^1^***

1. Institute of Nephrology, Zhong Da Hospital, Southeast University School of Medicine, Nanjing, Jiangsu, China
2. CT Bioscience CO. LTD, Changzhou, Jiangsu,China

**Correspondence to:** Bi-Cheng Liu, Institute of Nephrology, Zhong Da Hospital, Southeast Univ. School of Medicine, No. 87 Dingjiaqiao Rd, Nanjing, Jiangsu, China. Email: liubc64@163.com

| Symbol | 5' primer | 3' primer |
| --- | --- | --- |
| ACE | CCCAGGAGGATGTTTAAGGAG | CTCCAGCATCGACTTGTTCC |
| ACTB | AGTCCGCCTAGAAGCATTTG | CTGTCCACCTTCCAGCAGAT |
| AGT | ACAGCACCCTGGCTTTCA | TGGGAACAGACACTGAGGTG |
| B2M | TGTCTTTCAGCAAGGACTGG | AACTATCTTGGGCTGTGACAAA |
| BMP7 | CAACCACTGGGTGGTCAATC | CAGGTCTCGGAAGCTGACAT |
| C1GALT1 | TCATGCAAGGCATTCAGATG | CGGTCATAACCCAGCAAAGA |
| C1GALT1C1 | CACTCCGTTACGCTCCTGAC | TTAGCGAGACCAACGAGAGAA |
| CASP3 | AAGGTATCCATGGAGAACACTGA | TCCAGGGATATTCCAGAGTCC |
| CCL2 | AGGAAGATCTCAGTGCAGAGG | TCACAGCTTCTTTGGGACAC |
| CCL4 | GCTTCCTCGCAACTTTGTGG | TCACTGGGATCAGCACAGAC |
| CCL5 | CTGCTGCTTTGCCTACATTG | ACACACTTGGCGGTTCTTTC |
| CDH1 | AACCTCTGTGATGGAGGTCAC | TCAGGATCTTGGCTGAGGAT |
| COL3A1 | AGTGGACCTCCTGGCAAAG | TTACCTCGAGGCCCTGGT |
| COL4A3 | GGAGTAAAGGGCCAGAGAGG | TTTCCTTTCTCACCTGGATTTC |
| CTGF | AGGAGGCGTTGTCATTGGTA | CCTGGTCCAGACCACAGAGT |
| EGF | CCTTGGCCGTCTGGTTATAG | GCACCAGTACAACTTGTCAGTTAAG |
| FABP1 | CCTGGCTCTGCAGTTGGTA | CGTGAAGAGGGAGCTCTATTG |
| FN1 | CACCTCTGTGCAGACCACAT | GGTAAACAGCTGCACGAACA |
| GAPDH | ACTGCCACCCAGAAGACTGT | CAGGGATGATGTTCTGGAGAG |
| HAVCR1 | TCTCTACCTTTGTTCCTCCAATG | GTGAGCTGGTGGGTTCTCTC |
| HGF | CATGTCCTCCTGCATCTCCT | TGATTAGGGTAGTCTTTGCTGATTT |
| HPRT1 | ACGTCTTGCTCGAGATGTGA | AATCCAGCAGGTCAGCAAAG |
| ICAM1 | GCCCGAGCTCAAGTGTCTAA | CAGAGGTAGGTGCCCTCAAG |
| IGF1 | GCTGGTGGATGCTCTTCAGT | ACTCATCCACGATGCCTGTC |
| IL10 | CATCGATTTCTTCCCTGTGA | TGGCTTTGTAGATGCCTTTCT |
| IL18 | TCAACTCTCTCCTGTGAGAACAA | TCATGTCCTGGGACACTTCTC |
| IL1B | AGCTGATGGCCCTAAACAGA | GTGGTGGTCGGAGATTCGTA |
| IL6 | CCCAATAAATATAGGACTGGAGATG | GAGTTCATAGCTGGGCTCCT |
| IL8 | AAGACATACTCCAAACCTTTCCAC | AATTTCTGTGTTGGCGCAGT |
| ILK | TCTGCAGAAGAAGCCTGAAGA | GTACCTCCCGTGTCACCAGT |
| LCN2 | CAAGGAGCTGACTTCGGAAC | GACAGGGAAGACGATGTGGT |
| MMP2 | CTGGATCTACTCAGCCAGCAC | ATCCACTCGCTGGACATCA |
| MMP7 | TCCAACCTATGGAAATGGAGA | TGGATGTTCTGCCTGAAGTTT |
| MMP9 | GCTGGGCTTAGATCATTCCTC | GCCATTCACGTCGTCCTTAT |
| NFKB2 | GAAGATTGAGCGGCCTGTAA | AGGTGAACTGTTTGGAATCAGAC |
| NLRP3 | TGAAGTGCTGAAACAGCAGAG | AAAGACGACGGTCAGCTCAG |
| NPHS1 | AGGATTACGCCCTCTTCACA | TTTAATCCTGATGGAGGGTCA |
| NPHS2 | CAAGGTTGACCTTCGTCTCC | CCCAAATACAGGTCACTGAATC |
| OAZ1 | GGAACCGTAGACTCGCTCAT | TGAGCGTTTATTTGCACGAT |
| PDGFA | ACACGAGCAGTGTCAAGTGC | TTTGGCTTCTTCCTGACGTA |
| PDGFB | ACAAGACGGCACTGAAGGAG | GACGGACGAGGGAAACAATA |
| PLAUR | CGGCAGTCAATGAGGAAAGT | AATGGCCGCCAGTGTTAC |
| PODXL | CAGAGGCGACGACACGAT | TTTGTTAGATGAGTCCGTAGTAGTCTG |
| REN | TACCTTTGGTCTCCCGACAG | CTTCAGGCTTTCTCGGATTG |
| RPL27 | CTCTGGTGGCTGGAATTGA | TCTTTGATCTCTTGGCGATCT |
| S100A4 | GGGTCAGCAGCTCCTTTAGT | CACAAGTACTCGGGCAAAGA |
| SERPINE1 | CAACTTGCTTGGGAAAGGAG | ACTGGCCGTTGAAGTAGAGG |
| SMAD2 | CGAAATGCCACGGTAGAAAT | AGGGTGCCAGCCATATCTC |
| SMAD3 | CCCTGGCTACCTGAGTGAAGA | CTGCGTCCATGCTGTGGT |
| SMAD4 | CCATGGCCTTATTCATCCAC | TAGCAGCACTGGGACAACTG |
| SMAD7 | CAGATGCTGTGCCTTCCTC | CCAGGCTCCAGAAGAAGTTG |
| SNAI1 | AGTGCCTCGACCACTATGC | TGCAGCTCGCTGTAGTTAGG |
| SNAI2 | TGTGTGTCCAGTTCGCTGTAGTT | AGACCCGCTGGCAAGATG |
| ST6GALNAC2 | TGAAGAACTCCCTCGTCTCC | GCCGATCTCAGCATCACATA |
| SYNPO | GCCGCAAATCCATGTTTACT | CTCATCCGCTGTCTGTACCA |
| TF | AGTTTGCCCTGGTTTCACTG | GCACACATGGCAACACAGA |
| TFRC | CCACCATCTCGGTCATCAG | TGCTTTCTGAGGTTACCATCCT |
| TGFB1 | GAGCCCTGGACACCAACTAT | GTCCTTGCGGAAGTCAATGT |
| TGFB2 | CGAACCCAAAGGGTACAATG | GCAGCAAGGAGAAGCAGATG |
| TIMP1 | GTCCCAGATAGCCTGAATCCT | GAAAGATGGGAGTGGGAACA |
| TIMP2 | TGGCAACCCTATCAAGAGGA | CTTCTTTCCTCCAACGTCCA |
| TNF | GCCCGACTATCTCGACTTTG | ATGTTCGTCCTCCTCACAGG |
| TNFSF13 | GGAGTGCCAGGAGCACTAAC | AGTGGTGCAGAAGGGAAGAA |
| TP53 | AGGCCTTGGAACTCAAGGAT | TGAGTCAGGCCCTTCTGTCT |
| TWIST1 | CCTTCTCGGTCTGGAGGAT | CCAGAGTCTCTAGACTGTCCATTT |
| VEGFA | AAGGAGGAGGGCAGAATCAT | CACACAGGATGGCTTGAAGA |
| VIM | GCCAACCGGAACAATGAC | GTGAGGGACTGCACCTGTCT |
